# Deep‐Block: Large‐scale WGS Analysis for Alzheimer's Disease Risk Variant Detection Using Deep Learning

**DOI:** 10.1002/alz70856_106841

**Published:** 2026-01-08

**Authors:** Taeho Jo, Eun Hye Lee, Paula J Bice, Kwangsik Nho, Andrew J. Saykin

**Affiliations:** ^1^ Department of Radiology and Imaging Sciences, Indiana Alzheimer's Disease Research Center, Center for Neuroimaging, Indiana University School of Medicine, Indianapolis, IN, USA; ^2^ Center for Computational Biology and Bioinformatics, Indiana University School of Medicine, Indianapolis, IN, USA; ^3^ Department of Medical and Molecular Genetics, Indiana University School of Medicine, Indianapolis, IN, USA

## Abstract

**Background:**

The large‐scale WGS data from the Alzheimer's Disease Sequencing Project (ADSP) presents opportunities to identify novel genetic factors for Alzheimer's disease (AD). Advanced Artificial Intelligence (AI)‐based approaches may facilitate analysis of the WGS data. In this study, we developed Deep‐Block, a deep learning framework, to analyze ADSP R4 data, comprising 36,361 participant genomes. Our framework aims to identify robust AD‐associated genetic loci while retaining important biological context in an efficient manner. By integrating attention‐based neural networks, Deep‐Block captures intricate, non‐linear interactions among millions of genetic variants.

**Methods:**

We performed quality control on ADSP R4 WGS data (*N* = 36,361), retaining 9,956,115 SNPs and 36,329 participants (99.91%). We segmented the genome into 48,959 linkage disequilibrium (LD) blocks and imputed un‐called SNPs using k‐Nearest Neighbors. TabNet was used for feature selection, designating blocks exceeding 2.0 standard deviations above mean accuracy as high‐importance, yielding 3,040 blocks. A dual‐model approach (TabNet and Random Forest) computed importance scores for each SNP, identifying 4,869 genetic markers evaluated for their association with AD risk.

**Results:**

Deep‐Block yielded an AUC of 0.70 on an independent test set (*N* = 3,000), substantially outperforming randomly selecting SNPs (AUC=0.50) by 40%. Chromosome 19 harbored the largest number of high‐priority variants, including the known *APOE*‐related markers. In total, 95.6% of quality‐filtered SNPs were incorporated, indicating thorough genomic coverage.

**Conclusions:**

Deep‐Block is able to identify complex genomic features in AD. Model training and validation converged consistently, with cross‐validation demonstrating stable performance in AD risk prediction. By integrating LD‐based segmentation with deep learning approaches, the framework manages and addresses the complexity of large‐scale WGS data. Although high‐performance computing can expedite analysis, the method remains feasible in various research environments. Future directions include expansion of Deep‐Block to multi‐ethnic populations and incorporating multi‐omics data which may yield deeper insights into the genetic architecture of AD. Functional validation will be important to elucidate the influence of identified variants on AD pathogenesis.

